# Genetic Investigations in Turkish Idiopathic Pancreatitis Patients Show Unique Characteristics

**DOI:** 10.5152/tjg.2023.22773

**Published:** 2023-12-01

**Authors:** Hasan Baş, Selçuk Dişibeyaz, Erkin Öztaş, Yusuf Aydemir, Tuncer Temel, Oğuz Çilingir, Beyhan Durak Aras, Sevilhan Artan

**Affiliations:** 1 Department of Medical Genetics, Eskişehir Osmangazi University Faculty of Medicine, Eskişehir, Turkey; 2 Department of Medical Genetics, SBÜ Diyarbakır Gazi Yaşargil Training and Research Hospital, Diyarbakır, Turkey; 3 Department of Internal Medicine, Division of Gastroenterology, Eskişehir Osmangazi University Faculty of Medicine, Eskişehir, Turkey; 4 Division of Gastroenterology and Hepatology, Department of Pediatrics, Eskişehir Osmangazi University Faculty of Medicine, Eskişehir, Turkey

**Keywords:** Pancreatitis, PRSS1, SPINK1, CTRC, CFTR, idiopathic

## Abstract

**Background/Aims:**

Pancreatitis is one of the leading causes of digestive system-related hospital admissions, and it has a genetic background in a considerable portion of the patients. In this study, we aimed to investigate the genetic risk factors of idiopathic pancreatitis in Turkish patients and the contribution of copy number variations to the pathogenesis.

**Materials and Methods:**

Idiopathic pancreatitis is defined as failure to detect risk factors despite comprehensive clinical assessments. Next-generation sequencing and multiple ligand-dependent probe amplification of *PRSS1*, *SPINK1*, *CTRC*, and *CFTR* were performed. For further genotype–phenotype correlations, patients were also questioned for the age of onset, family history, and pancreatic divisum.

**Results:**

A total of 68 idiopathic pancreatitis cases were enrolled. Variants with potential clinical significance of *PRSS1* were identified in 13.4%, *SPINK1* in 6.3%, *CTRC* in 4.7%, and *CFTR* in 26.5% of the patients. No copy number variants were seen in any of these genes. At least 7.4% of the participants had complex genetic etiology involving 2 genes.

**Conclusions:**

At least 42.6% of the participants had a potential genetic risk factor. Five novel genetic variants were identified, and distinctive genetic risk factors of Turkish population were shown. The results showed that genetic etiology was frequent in pancreatitis and it was even more prominent in patients with early-onset disease. Considering that genetic risk factors may be informative for decision-making in the treatment options in addition to providing extensive prognostic value and familial genetic consultation; clinicians need to be more eager to offer genetic tests to pancreatitis patients.

**Main Points**

- At least 42.6% of the participants had potential genetic risk factors.
- Copy number variations were not identified in the cohort.
- Common c.180C>T (p.G60G) variant of *CTRC* was significantly associated with pancreatitis.
- Early-onset pancreatitis was more prominently associated with the presence of genetic risk factors.

## Introduction

Pancreatitis is one of the leading causes of digestive system-related hospital admissions and leads to a significant amount of morbidities, mortalities, and socioeconomic burden.^1^ Pancreatitis is interpreted in 3 subgroups named acute pancreatitis (AP), recurrent AP (RAP), and chronic pancreatitis (CP) according to the onset and frequency of the symptoms.^2^ Even though these subgroups are known as separate clinical entities, some of the previous studies hypothesized that they may be the different stages of a continuum disease spectrum.

The etiology of pancreatitis is diverse. Alcohol and biliary factors are the most common inducers of AP/RAP, whereas chronic alcohol abuse and continuous smoking are the leading causes of CP.^3^ Despite the investigations for other most frequent etiological factors including hypertriglyceridemia, hypercalcemia, infections, or obstructive masses, the cause of pancreatitis cannot be determined in 10%-30% of events and they are called “idiopathic pancreatitis.”^4^ It is known that genetic contributions are identified in a considerable portion of those patients with so-called idiopathic pancreatitis. 

The majority of the pancreatitis-associated genes, like *PRSS1*, *SPINK1*, and *CTRC*, encode proteins involved in trypsin-dependent digestion, whereas *CFTR* variations lead to pancreatitis by disrupting CFTR channel functions.^1^ Besides these 4 major genes, *CPA1*, *CEL-HYB* hybrid allele, an inversion in *CTRB1-CTRB2* locus, *CLDN2*, *CASR*, and several other genes were recently reported in pancreatitis.^1, 5^ Studies focusing on variants of these genes in pancreatitis showed that genetic risk factors can be found in 30%-60% of idiopathic RAP and 12%-43% of idiopathic CP.^4^ Most of the previous studies in the literature only focused on sequence variants and there are only a limited number of studies which revealed that copy number variants were linked to disease in up to 5% of patients.^6, 7^ Therefore, a concern occurred for possible undiagnosed patients. Also, genetic variants were vastly different in distinct regions of the world, and the single paper disclosing the genetic risk factors of pancreatitis in Turkish patients only evaluated the most frequent pathogenic variants.^8, 9^ In this study, we aimed to investigate the genetic risk factors of pancreatitis in the Turkish population and the contribution of copy number variations to the pathogenesis of idiopathic pancreatitis. 

## Materials and Methods

### Study Design and Participants

The study was approved by Eskişehir Osmangazi University Non-interventional Clinical Researches Ethics Committee (Approval No: 04, Date: August 6, 2019). Each patient provided informed consent. The clinical information of the patients was obtained from the patients themselves and their hospital records. Both pediatric and adult patients were included. The diagnoses of AP, RAP, or CP were determined according to the latest guidelines.^2, 10^ Idiopathic pancreatitis was defined as failure to detect risk factors despite comprehensive clinical assessments. Therefore, patients with biliary pancreatitis, alcohol abuse (≥40 g/day or ≥300 g/week), heavy smoking (>20 cigarettes/day), hyperlipidemia, hypercalcemia, trauma, drug use, and autoimmune pancreatitis were excluded.^3, 10^ Because the contribution of pancreas divisum to the pathogenesis of pancreatitis is still controversial, patients with pancreas divisum were not excluded, but they were evaluated separately to see if it acts as a risk factor in the presence of certain genetic variants as previously reported.^11-13^

For further clinical investigations, patients were questioned for the age of onset and family history. The presence of pancreas divisum was assessed. The age of symptom onset was defined as the age at the time when the first abdominal pain consistent with pancreatitis appeared. Positive family history was determined when there was another family member with pancreatitis in the 3-generation pedigree chart. The diagnosis of pancreas divisum was evaluated by magnetic resonance cholangiopancreatography (MRCP) or endoscopic retrograde cholangiopancreatography (ERCP). If these were not available or gave inconclusive results, the patients were excluded from this part of the study. 

For genetic testing, next-generation sequencing (NGS) and multiplex ligation-dependent probe amplification (MLPA) of *PRSS1, SPINK1, CTRC,* and *CFTR* genes were performed. The NGS variants were classified according to American Collage of Medical Genetics (ACMG) criteria and variants of uncertain significance (VUS), likely pathogenic and pathogenic variants were presented as “variants with potential clinical significance.” A common synonym variant of *CTRC*, c.180C>T (p.G60G), was interpreted separately as it was previously reported to be a risk factor.^14^ To compare the frequency of this variant with Turkish controls, whole-exome sequencing data from the Intergen Genetic Diagnosis Center were used as a control. The SALSA MLPA Probemix P242 Pancreatitis and SALSA MLPA Probemix P091 CFTR probes were used to determine the copy number variants of these 4 genes. 

### Statistical Analysis

Statistical analyses were performed on IBM’s Statistical Package for the Social Sciences version 21.0 (IBM Corp., Armonk, NY, USA). Summary statistics of categorical variables were shown as mean with standard deviation (SD), median with interquartile range (IQR), or frequency count with percentage. Fisher exact, Yates, or Pearson chi-square test was used to compare categorical variables and between-group differences, a *P*-value of <.05 was considered as statistically significant.

## Results

### Patient Characteristics

A total of 68 patients with idiopathic pancreatitis were enrolled. Twenty-eight (41.2%) participants were female and 40 (58.8%) were male. Thirteen (19.1%) had AP, 26 (38.2%) had RAP, and 29 (42.6%) had CP. Sixteen (23.5%) patients were children, and 52 (76.5%) were adults at the time of enrollment (minimum: 5, maximum: 85, mean: 35.9, SD: ±19.3, median: 34.5, IQR: 30). Twenty-three (33.8%) had symptom onset during childhood (≤18), and 45 (66.2%) patients during adulthood (minimum: 3, maximum: 82, mean: 30.7, SD: ±20.0, median: 27.5, IQR: 31). Only 11 (16.2%) participants had a family history of pancreatitis. Out of 64 evaluated patients, 11 (17.2%) had pancreas divisum (Table 1).

### Molecular Results

**Figure 1.**
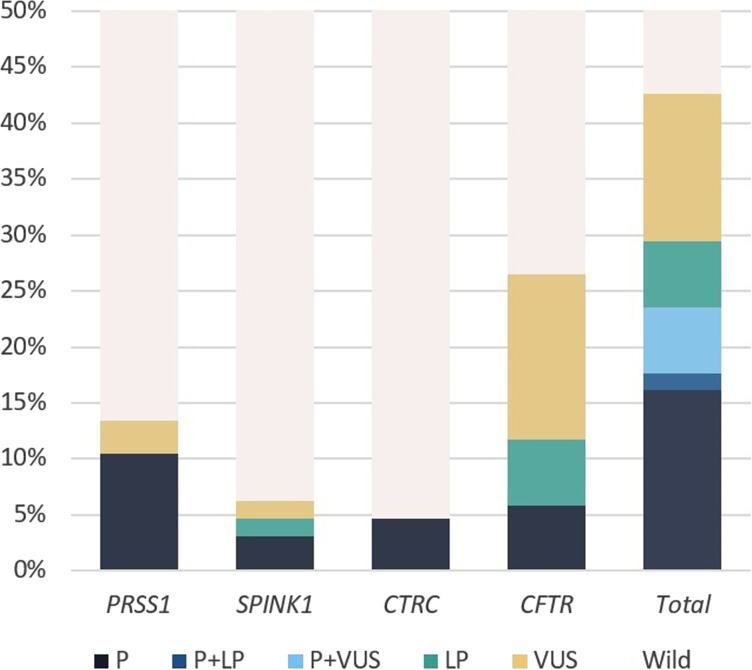
The percentages and classifications of identified variants in each gene and total.

Out of 68 participants, NGS of *PRSS1* was available in 67, *SPINK1* in 64, *CTRC* in 64, *CFTR* in 68, and MLPA was performed in 64. Of these, variants with potential clinical significance of *PRSS1* were identified in 9 (13.4%), *SPINK1* in 4 (6.3%), *CTRC* in 3 (4.7%), and *CFTR* in 18 (26.5%) by NGS. No copy number variations were seen in the MLPA of all 4 genes. So, potential genetic risk factors were found in at least 29 (42.6%) participants (Figure 1). At least 5 (7.4%) patients had complex genetic etiology involving 2 genes: 2 *PRSS1* patients, 1 with *SPINK1*, and 2 with *CTRC* variants also had *CFTR* variants. 

The variant frequency varied little according to the diagnosis, other than pathogenic *PRSS1* variants which were more common in the CP group (AP: 0.0%, RAP: 7.7%, CP: 17.2%). Variants with potential clinical significance were identified in at least 44.8% of CP, 38.4% of RAP, and 46.2% of AP patients (Table 2). 

The detailed characteristics of variants and the participants carrying them were listed in Table 3. There were 5 different *PRSS1* variants, 2 were VUS and 3 were pathogenic. c.365G>A; p.(R122H) was the most common, and it was present in more than half of *PRSS1* patients. Except for one, clinical findings of participants with pathogenic *PRSS1* variants were apparent in pediatric ages, and the earliest symptom onset was seen in patient #4 who had an additional *CFTR* variant. Patient #8 noticed his first symptoms at 71, but he had already developed CP. The majority of the patients (5/7, 71.4%) with pathogenic *PRSS1* variants had a positive family history. Molecular tests were not available for these symptomatic family members, but 12-year-old asymptomatic son of patient #27 and 39-year-old asymptomatic father (whose mother was symptomatic at 7) of #39 were carriers of the defined variants. Both patients with *PRSS1* VUS had no family history, and segregation analysis revealed 30-year-old asymptomatic son of patient #12 also had the variant. 

Two *SPINK1* variants were known pathogenic variants, the novel frameshift in patient #38, c.162delT p.(N56Mfs*39), was classified as likely pathogenic, and the novel missense variant in patient #33, c.181T>C p.(C61R), was classified as VUS. Both patients did not have a family history of pancreatitis, and segregation analysis could not be performed. 

All 3 *CTRC* patients had the same variant, but only 1 with very early-onset disease, patient #31, had the variant in the homozygous state, besides a *CFTR* variant. The 35-year-old asymptomatic parents of this patient were heterozygous, and the *CFTR* variant was inherited from the mother. Family history was positive in the other 2, genetic testing was performed on the siblings of patient #25, and both of his symptomatic 51-year-old brother and asymptomatic 41-year-old sister had the same variant.

Out of 14 *CFTR* variants we identified, 4 were pathogenic, 3 were likely pathogenic, and 7 were VUS. Three of these variants, c.869+2T>C, c.2605A>G; (p.I869V), c.3401C>T; (p.T1134I) were novel and while both missense variants were classified as VUS; splice site variant c.869+2T>C was pathogenic (Table 4). Only patients #42 and #43 with isolated *CFTR* variants had family histories, but segregation analyses were not available.

Among the 11 patients with idiopathic pancreatitis with pancreas divisum, 4 (36.4%) had *CFTR* variants, and variants of other genes were not detected (Table 4). The *CFTR* variants were present in 13 (24.5%) patients without pancreas divisum. In addition, 7 (20.0%) patients with no clinically significant variants had pancreas divisum.

**Figure 2.**
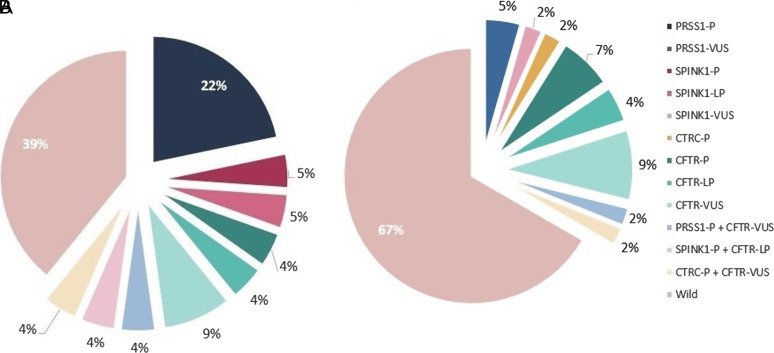
Variant frequency by the age of symptom onset. (A) Variant frequency in patients with childhood onset. (B) Variant frequency in patients with adulthood onset. LP, likely pathogenic; P, pathogenic; VUS, variant of uncertain significance.

Potential genetic risk factors were found in 60.9% of the patients with childhood-onset symptoms and in 33.3% of the patients with adult-onset symptoms. Pathogenic *PRSS1* and *SPINK1* variants were predominantly present in patients with childhood-onset disease (Figure 2). Potential genetic risk factors were identified in 81.8% of the patients who had a family history and in 36.4% of the patients who did not have one.

The common c.180C>T variant of *CTRC* was identified in 17 participants (heterozygous in 14, homozygous in 3, allele frequency: 15.6%). The allele frequency of the variant was 9.4% (393/4186) in our in-house exome sequencing data, so the variant was statistically more frequent in idiopathic pancreatitis patients (*P *= .027). It was also statistically confirmed that the variant was more frequent in idiopathic CP patients compared to the controls (*P *= .014). The variant was more common in idiopathic CP than idiopathic RAP + AP group, but the *P*-value was not statistically significant (*P = *.239) (Table 5). 

## Discussion

Various frequencies of genetic variants have been found to contribute to the pathogenesis of pancreatitis in studies so far. As well as the ethnic characteristics of the populations, different outcomes were also related to the demographic/clinical characteristics of the participants, which genetic testing methods were used, and which variants the researchers deemed appropriate to report. In this study, *PRSS1, SPINK1, CTRC, *and *CFTR* genes were analyzed by NGS and MLPA to detect both sequence and copy number variants in children and adults with idiopathic pancreatitis and pathogenic/likely pathogenic variants and VUS were reported.

### *PRSS1* Variants

*PRSS1* is the first gene associated with pancreatitis, and the most common *PRSS1* variants, p.R122H and p.N29I, are expected to be penetrant in as high as 90% of the carriers.^15^ The highest *PRSS1* variant frequencies, 31.3% and 30%, were reported in INSPPIRE studies by Giefer et al^15^ in 240 and Dike et al^8^ in 333 children patients with RAP/CP respectively, and some smaller studies reported that none of their patients with idiopathic CP or RAP had *PRSS1* variations.^13^ *PRSS1* variants were defined in 13.4% of our participants, and the most common was p.R122H, consistent with the previous reports. The higher rate of Giefer et al^15^ and Dike et al^8^ might be attributed to the demographics of their participants since the studies were performed on pediatric patients.^13^ The* PRSS1* variant frequency of our patients whose symptoms started during the pediatric period (26.1%) was comparable to those studies, and our overall *PRSS1* variant frequency was similar to large Asian studies planned in both adults and children.^16^ The highest CNV rate of *PRSS1* was reported as 5.1% in a French study of patients with CP, but no CNVs were detected in the present study.^7^

### *SPINK1* Variants

*SPINK1* was previously stated to be less risky and considered to cause pancreatitis in hardly 1% of pathogenic variant carriers; however, recent Asian studies make this information controversial.^16, 17^ It is stated that pathogenic *SPINK1* variants are usually found in patients with other genetic/environmental risk factors; therefore, it is unclear whether pathogenic *SPINK1* variants cause all types of pancreatitis by themselves or act like cofactors.^18^ *SPINK1* variants are especially common in Asia, Wang et al^19^ and Liu et al^20^ reported variant frequencies of 57.3% and 56.2% in their CP patients, respectively. The *SPINK1* variant rate and profile of our study have differed from these previous researches. The variant frequency was 6.3% and 2 novel variants, c.162delT (p.N56Mfs*39) and c.181T>C (p.C61R), were defined. Large deletions of *SPINK1* were reported in small case reports, but they were not reported in larger studies, and no copy number variant of *SPINK1 *was identified in this study too.^6, 7^

### *CTRC* Variants

Initial studies indicated that *CTRC *variants were rare causes of pancreatitis, but later, it was found that a common silent variant, c.180C>T, is present in up to 30% of CP patients and may be a risk factor for pancreatitis.^14, 21^ While most studies showed that other *CTRC* variants were present in less than 5%, the highest rate was reported by Grabarczyk et al,^22^ 8.8% of their patients with idiopathic CP had pathogenic *CTRC* variants and 49% had common c.180C>T.^4, 7, 16^ Consistent with most of these studies, *CTRC* variants were present in 4.8%, and c.180C>T was seen in 26.6% of the participants with an allele frequency of 15.6%. There was a statistically significant difference between the allele frequency of it in both idiopathic pancreatitis patients versus controls (*P *= .027) and idiopathic CP patients versus controls (*P *= .014). It was also shown that it was more frequent in the idiopathic CP than the idiopathic RAP + AP group, but this correlation has not been proven statistically (*P = *.239). These results were consistent with previous research remarking that c.180C>T variant is associated with CP, and pancreatitis in general.^14, 22^ Nevertheless, the variant being more common in the CP than in the RAP + AP group supported the conclusion of LaRusch et al^14^ that this variant might play a role in disease progression from AP/RAP to CP. Just like *SPINK1*, CNVs of *CTRC* were shown in small case reports, but they were not identified in the only previous big cohort evaluating them.^7, 23^ We did not find CNVs of *CTRC* in our participants either.

### *CFTR* Variants

In addition to cystic fibrosis, pathogenic variants of *CFTR* are also responsible for non-classic cystic fibrosis phenotypes like congenital vas deferens agenesis, chronic rhinosinusitis, and pancreatitis.^24^ Unlike variants causing severe impairment in *CFTR* channel function and resulting in classical cystic fibrosis, milder variants or variants disturbing bicarbonate permeation lead to a higher risk for pancreatitis, and even variants which are not associated with classical cystic fibrosis, like p.R117H or p.L997F, are reported to increase the risk for pancreatitis.^1, 23, 25^ Because of this, interpreting *CFTR* variants in pancreatitis is challenging and various studies revealed vastly different outcomes.^4, 15, 16, 22^ In this study, we classified *CFTR* variants by their potential pathogenicity for pancreatitis instead of classical cystic fibrosis and it was found that 26.5% of our participants had *CFTR* variants with potential clinical significance. Like previous studies, most patients had milder variants and none had the most common *CFTR* variant in classical cystic fibrosis, p.F508del.^1, 25^ We also identified 3 novel *CFTR* variants c.869+2T>C, c.2605A>G; (p.I869V), c.3401C>T; (p.T1134I), but no segregation or functional analyses were available for these. The CNVs of *CFTR *in pancreatitis were also rarely studied, and an anomaly was once reported by Sofia et al^5^ in 1 of their 80 CP patients. None of our patients had CNVs of *CFTR*.

### Complex Genetic Etiology

Pancreatitis is a multifactorial disease with complex genetic etiology, and most studies revealed that a portion of patients had multiple genetic risk factors. Similar to previous studies, complex genetic etiology was present in at least 5.9% of patients; 2 *PRSS1*, 1 *SPINK1*, and 2 *CTRC* patients also had *CFTR* variants.^7^ Previous studies reported a possible synergist effect of *SPINK1* and *CFTR *variants on the development of CP.^26^ The majority of our *CFTR* patients did not have *SPINK1* variants and vice versa, but we had a very limited number of patients and it was not possible to come up with any conclusion on this matter.

### Age of Symptom Onset, Family History, and Pancreas Divisum

Just as on many other disorders, we found that early-onset pancreatitis was associated with the presence of genetic etiology. In our study, 60.9% of patients with childhood-onset and 33.3% with adult-onset disease had potential genetic risk factors. The majority of patients with pathogenic *PRSS1* and *SPINK1* variants had the childhood-onset disease, only patient #8 with a highly penetrant p.R122H variant of *PRSS1* realized his first symptoms at 71. But since the feeling of pain or suspecting of a serious disease during an abdominal pain attack is such subjective issues, the patient may have earlier disease onset or might have had subclinical markers before. As expected, patients with complex genetic risk factors had earlier disease onset, but interestingly, most patients with pathogenic *CFTR* variants developed their symptoms after the fifth decade. Two patients with the late-onset disease (patients #6 and #64) had variants (c.3140-26A>G, c.3154T>G) responsive to CFTR modulator therapies, and considering this, clinicians should keep in mind that the genetic testing would also be informative for treatment options of patients with late-onset disease.^27, 28^

The presence of potential genetic risk factors in 81.8% of patients with a family history and 36.4% of others emphasizes that genetic testing should not be limited to patients with a family history. Unfortunately, segregation analysis could not be performed for each patient carrying genetic variants; thus, we do not know if negative family histories on patients with genetic risk factors are caused by reduced penetrance or *de novo* mutations.

There are conflicting opinions on the association between pancreas divisum and the development of pancreatitis. Although some studies show pancreas divisum is an individual risk factor for pancreatitis, some other studies indicate that it acts as a cofactor to other risk factors, including genetic variants.^11, 12^ Although we did not have a control group to determine the frequency of pancreas divisum in our local healthy population; it is known that the frequency of pancreas divisum is 4%-10%, and in a recent large study, it was found that it was present in 4.6% of 1158 Turkish individuals.^29, 30^ Pancreas divisum was more frequent in our patients with a frequency of 17.2% and the frequency of it was even higher (20%) in patients with no significant genetic risk factor. These results support that pancreas divisum may individually create a risk for pancreatitis, but larger studies with proper healthy control groups are needed for confirmation. Bertin et al^12^ suggested that pancreas divisum especially confers risk when it accompanies *CFTR* variants. The frequency of *CFTR* variants was 36.4% in our pancreas divisum patients and 24.5% in patients without pancreas divisum. These results hint at a possible association, but since we had a small group of patients, we could not statistically confirm the link between pancreas divisum and *CFTR*.

In conclusion, in this study of genetic risk factors in Turkish idiopathic pancreatitis patients, we found that at least 42.6% of our participants had potential genetic risk factors. c.365G>A; p.(R122H) variant of *PRSS1* was the most common variant identified in this study, especially in idiopathic CP patients with early-onset disease, and in general, *CFTR* variants were most frequent. No copy number variations were identified, and 5 single-nucleotide variants were novel (*SPINK1*: c.162delT, c.181T>C; *CFTR*: c.869+2T>C, c.2605A>G, c.3401C>T). We also found that the common c.180C>T variant of *CTRC* was significantly associated with pancreatitis. The biggest limitation of this study was that our participants were a relatively small group of patients, partly due to the COVID-19 pandemic, and segregation analyses could not be completed mainly because of the same reason. Another limitation is that we only performed genetic tests in 4 genes associated with pancreatitis, but other recently described pancreatitis-related genes were not analyzed, so some risk factors in these genes could have been missed. In this study, distinctive genetic risk factors of Turkish pancreatitis patients were shown but larger and long-term studies are still needed to search for other unique genetic variants, investigate pancreas divisum/pancreatitis genetics and understand clinical characteristics of patients with genetic risk factors. 

## Figures and Tables

**Table 1.** Clinical and Demographic Characteristics of the Study Group

	Diagnosis			Sex		Age at Enrollment		Age of Symptom Onset		Family History		Pancreas Divisum	
	AP	RAP	CP	F	M	C	A	C	A	+	−	+	−
Number	13	26	29	28	40	16	52	23	45	11	55	11	53
Percentage	19.1%	38.2%	42.6%	41.2%	58.8%					16.2%	80.9%	17.2%	82.8%
Mean						Mean: 35.88 Median: 34.50		Mean: 30.74 Median: 27.50					

+, present; −, absent; A, adulthood; AP, acute pancreatitis; C, childhood; CP, chronic pancreatitis; F, female; M, male; RAP, recurrent acute pancreatitis.

**Table 2.** Variant Frequency in Each Diagnostic Subgroup

	CP			RAP			AP		
	P	LP	VUS	P	LP	VUS	P	LP	VUS
***PRSS1***	5/29 (17.2%)	0/29 (0.0%)	0/29 (0.0%)	2/26 (7.7%)	0/26 (0.0%)	1/26 (3.8%)	0/12 (0.0%)	0/12 (0.0%)	1/12 (8.3%)
***SPINK1***	1/26 (3.8%)	1/26 (3.8%)	0/26 (0.0%)	1/26 (3.8%)	1/26 (3.8%)	1/26 (3.8%)	0/12 (0.0%)	0/12 (0.0%)	0/12 (0.0%)
***CTRC***	2/26 (7.7%)	0/26 (0.0%)	0/26 (0.0%)	0/26 (0.0%)	0/26 (0.0%)	0/26 (0.0%)	1/12 (8.3%)	0/12 (0.0%)	0/12 (0.0%)
***CFTR***	1/29 (3.5%)	1/29 (3.5%)	4/29 (13.8%)	1/26 (3.8%)	1/26 (3.8%)	5/26 (19.2%)	2/13 (15.4%)	2/13 (15.4%)	1/13 (7.7%)
**Total**		13/29 (44.8%)			10/26 (38.4%)		
6/13 (46.2%)	

AP, acute pancreatitis; CP, chronic pancreatitis; LP, likely pathogenic; P, pathogenic; RAP, recurrent acute pancreatitis; VUS, variant of uncertain significance.

**Table 3.** Characteristics and Classifications of the Identified Variants with Respect to the Clinical Features of the Patients

Variant	MAF (%)	CADD	REVEL	phyloP	MT	Classification	Patient	Zygosity	Diagnosis	Onset Age	Enrollment Age
*PRSS1*: NM_002769.4											
c.62A>C; p.(D21A)	N/A	23.2	0.674	4.039	DC	Pathogenic	#37	Heterozygous	CP	14	21
c.310C>G; p.(L104V)	N/A	7.443	0.36	-0.35	DC	VUS	#12	Heterozygous	AP	69	70
c.364C>T; p.(R122C)	0.001988	18.36	0.515	0.068	DCA	Pathogenic	#39	Heterozygous	RAP	7	10
c.365G>A; p.(R122H)	0.001062	7.883	0.511	0.14	DCA	Pathogenic	#4	Heterozygous	RAP	5	10
							#8	Heterozygous	CP	71	73
							#15	Heterozygous	CP	9	18
							#28	Heterozygous	CP	17	41
							#35	Heterozygous	CP	14	18
c.592-4C>T	0.003182	N/A	N/A	N/A	N/A	VUS	#54	Heterozygous	RAP	28	35
*SPINK1*: NM_003122.4											
c.101A>G; p.(N34S)	0.9028	0.123	0.199	-3.751	P	Pathogenic	#50	Heterozygous	CP	5	22
c.162delT; p.(N56Mfs*39)	N/A	N/A	N/A	N/A	N/A	Likely pathogenic	#38	Heterozygous	CP	9	12
c.181T>C; p.(C61R)	N/A	23.8	0.722	4.349	DC	VUS	#33	Heterozygous	RAP	23	25
c.194+2T>C	0.03033	24.3	N/A	4.349	DC	Pathogenic	#49	Heterozygous	RAP	13	19
*CTRC*: NM_007272.3											
c.703G>A; p.(V235I)	0.1036	25.4	0.797	3.662	DC	Pathogenic	#21	Heterozygous	CP	28	33
							#25	Heterozygous	CP	20	38
							#31	Homozygous	AP	4	5
*CFTR*: NM_000492.3											
c.274-6T>C	0.04613	N/A	N/A	N/A	N/A	VUS	#26	Heterozygous	RAP	40	41
c.358G>A; p.(A120T)	0.01381	23.4	0.787	9.516	DC	Likely pathogenic	#49	Heterozygous	RAP	13	19
c.869+2T>C	N/A	28.5	N/A	7.544	DC	Pathogenic	#36	Heterozygous	RAP	7	11
c.1516A>G; p.(I506V)	0.03573	23.7	0.57	8.79	DCA	VUS	#4	Heterozygous	RAP	5	10
c.2605A>G; p.(I869V)	N/A	10.65	0.316	-0.006	P	VUS	#21	Heterozygous	CP	28	33
c.2856G>C; p.(M952I)	0.007957	23.6	0.817	9.755	DC	Pathogenic	#22	Heterozygous	AP	43	44
c.2991G>C; p.(L997F)	0.2222	23.7	0.625	1.651	DC	Likely pathogenic	#29	Heterozygous	CP	19	27
							#41	Heterozygous	RAP	11	17
c.3140-26A>G	0.00485	N/A	N/A	N/A	N/A	Pathogenic	#64	Heterozygous	AP	59	59
c.3151A>G; p.(I1051V)	0.003552	26.4	0.661	9.265	DC	Likely pathogenic	#24	Heterozygous	AP	45	46
c.3154T>G; p.(F1052V)	0.06282	29.9	0.948	7.967	DC	Pathogenic	#6	Heterozygous	CP	52	55
c.3401C>T; p.(T1134I)	N/A	25	0.756	5.944	DC	VUS	#8	Heterozygous	CP	71	73
c.4220T>C; p.(M1407T)	N/A	23.1	0.703	7.323	DC	VUS	#31	Heterozygous	AP	4	5
c.2260G>A; p.(V754M)	0.182	11.14	0.518	0.1772	P	VUS	#68	Heterozygous	CP	44	60
IVS8-5T	N/A	N/A	N/A	N/A	N/A	VUS	#41	Heterozygous	RAP	11	17
							#43	Heterozygous	RAP	39	41
							#52	Heterozygous	CP	19	32
							#58	Heterozygous	RAP	4	12

AP, acute pancreatitis; CADD, combined annotation-dependent depletion; CP, chronic pancreatitis; DC, disease causing; DCA, disease causing automatic; MAF, minor allele frequency (gnomAD); MT, MutationTaster V2; N/A, not available; phyloP: phyloP 100way vertebrate; P, polymorphism; RAP, recurrent acute pancreatitis; REVEL, rare exome variant ensemble learner; VUS, variant of uncertain significance.

**Table 4.** Variant Frequencies in Patients with/Without Pancreas Divisum

	*PRSS1*	*SPINK1*	*CTRC*	*CFTR*	Total
Pancreas divisum +	0/11 0.0%	0/11 0.0%	0/11 0.0%	4/11 36.4%	4/11 36.4%
Pancreas divisum −	9/52 17.3%	4/49 8.2%	1/49 2.0%	13/53 24.5%	23/53 43.4%

**Table 5.** The Allele Frequency of c.180C>T (p.G60G) Variant of *CTRC *in the Participants and the Controls

	IP	Controls	ICP	Controls	ICP	IRAP + IAP
Allele frequency	20/128 (15.6%)	393/4186 (9.4%)	11/52 (21.2%)	393/4186 (9.4%)	11/52 (21.2%)	9/76 (11.8%)
*P*	.027		.014		.239	

IAP, idiopathic acute pancreatitis; ICP, idiopathic chronic pancreatitis; IP, idiopathic pancreatitis; IRAP, idiopathic recurrent pancreatitis.
